# GHB, Chemsex and Chemical Submission: Investigating the Role of Sexuality on Victim Empathy and Blame Attribution in Drug-Facilitated Sexual Assault against Men

**DOI:** 10.3390/bs14100913

**Published:** 2024-10-08

**Authors:** Harrison Lee, Danielle Labhardt, Dominic Willmott

**Affiliations:** 1 Department of Psychology, Manchester Metropolitan University, Manchester M15 6GX, UKd.labhardt@mmu.ac.uk (D.L.); 2 Department of Criminology, Sociology and Social Policy, Loughborough University, Leicestershire LE11 3TU, UK; 3 Department of Social Psychology, SWPS University, 53-238 Wrocław, Poland

**Keywords:** male sexual victimisation, chemsex, gamma-hydroxybutyrate (GHB), victim empathy, blame attribution, victim blame, male rape, sexuality, men who have sex with men (MSM)

## Abstract

Literature is sparse regarding men’s attitudes towards male sexual assault and the role that the sexuality of those involved may have. Despite the high prevalence of chemsex and GHB (gamma-hydroxybutyrate) participation among men who have sex with men, no study has yet investigated attitudes towards such. Utilising a community sample of 141 UK men, participants were randomly assigned into one of six conditions based on victim sexuality (heterosexual or homosexual) and the drug used present during the sexual assault (chemsex, chemical submission, or no drugs). All participants completed the Male Rape Victim and Perpetrator Blaming Scale and Victim-Blaming Empathy Scale to measure victim-blame and empathy attributions. Results of a two-way MANOVA revealed a significant difference between participant gender and empathic ratings, with heterosexual participants significantly less likely to empathise than their homosexual counterparts. A non-significant difference was observed between the conditions alongside a non-significant interaction. Nevertheless, results indicate that victims in the chemsex condition, along with heterosexual victims, encountered the greatest victim-blaming attributions and the lowest rates of participant empathy overall. Findings overall appear to indicate a general decline in victim-blame attitudes towards men who have sex with men, though a level of uncertainty was apparent among the sample. Implications and limitations of the work are discussed alongside the importance of future research and psychoeducation interventions.

## 1. Introduction

Prior research suggests that men who have sex with men (MSM) are significantly more likely to participate in risk-taking behaviours [1], including polysubstance use [2] and anonymous sex with multiple sexual partners [3]. MSM are twice as likely to use drugs and alcohol as straight men and three times more likely to use illicit substances [4]. The widely accepted practice of casual drug use among MSM is reflected in what has become known as ‘chemsex’ [5]. Chemsex is the contraction of ‘chemical sex’ and refers to the voluntary taking of substances to enhance and/or facilitate sexual encounters [6]. Its prevalence amongst MSM groups varies, ranging from 6% [7] to 90% [8]. On the contrary, studies indicate prevalence ranges from 1% [9] to 12% [9] among heterosexual men. Although chemsex is significantly more common with MSM, Incera-Fernandz et al. [9] found that heterosexual and homosexual men aged below 35 were more likely to participate in this practice. While the study was carried out among a Spanish sample, Rosner et al. [10] also point to age being an important factor in chemsex use, as is found for drug use more generally [11,12], and additional research illustrates the rise of this phenomenon in recent years, regardless of sexuality [13,14]. As chemsex is a relatively contemporary phenomenon, research is somewhat scarce, albeit in agreement that greater insights and awareness are needed among the UK public [15,16]. Chemsex decreases risk perception [17,18] and is associated with casual sex with multiple partners [19], unconventional sexual practices [20], and an increase in STI contraction, including HIV, hepatitis C and Shigella flexneri [21,22]. Recently, the European Centre for Disease Prevention and Control [23] stated that Chemsex could further facilitate the transmission and sudden rise of the Monkeypox virus amongst MSM groups. Indeed, Tomkins and colleagues’ [24] systematic review shows an association between chemsex and addictive, depressive and anxious symptoms amongst MSM, and Miltz et al. [25] found a similar association amongst heterosexual men. Chemsex is linked with severe significant health consequences, with Public Health England [4] signalling its aim to reduce chemsex amongst MSM, echoing calls for the importance of further research. According to the Minority Stress Theory, MSM may (mis)use substances as a coping strategy for the frequent experiences of external prejudice, internalised homophobia and rejection expectations [26]. The Cognitive Escape Model lends credence to the theory that MSM use drugs to escape the pressures of reality and the stigma associated with their sexual orientation [27]. It should be noted that very few scholars have applied this theory and model to MSM compared to other minority groups. However, Preston et al. [28] found that MSM with higher rates of perceived stigma and rejection have a higher chance of engaging in risky sexual behaviour, including Chemsex, lending support to the theoretical stance.

Substances often used in chemsex include mephedrone [29], butyl nitrites (‘poppers’) [30], MDMA [31] and, most alarmingly for professionals, Gamma-Hydroxybutyrate [GHB] [31]. GHB is a central nervous system depressant with hypnotic and euphoric properties [32]. The drug has been labelled problematic [33] as it is cheap, easily accessible and often heralded as safe [34,35]. The British Crime Survey [36] found that 0.1% of the British population reported using GHB, yet a study conducted by Buzzfeed News [37] found that 54% of MSM had taken GHB. The survey revealed worrying figures, with all GHB users knowing someone who had been raped or sexually assaulted when using the drug, and 25% had been assaulted. Indeed, 62.5% of users reported issues while under the influence, including addiction, hospitalisation, unconsciousness and sexual assault (SA). 

Moreover, 27% of MSM report knowing someone who has died from consuming GHB. The drug is often named the ‘date-rape’ drug [38]; its effects of severely incapacitating individuals, given its odourless and colourless properties [39], make individuals under the influence of GHB more vulnerable to crime [40]. Buzzfeed News [37] found that 47% of MSM suspect or know they had been given GHB without their consent, with 18% reporting experiencing a deliberate overdose. Giving someone a substance without their consent, often termed ‘spiking’ or ‘chemical submission’, is an important gendered issue, yet mainstream media and academic literature predominately focus on female victimisation due to the rates at which women are disproportionally affected by gender-based violence [41,42,43,44]. 

However, a YouGov [45] study found that one in five men report knowing or having firsthand experience of men being spiked. Swan et al.’s [46] study reflected this finding that in their sample, 22% of spiking victims were male, with 16% reporting sexual assault (SA) as the primary motive. Shockingly, Swan et al.’s [46] study found that 37.2% of men were thought to enjoy being spiked (though for the broader context surrounding male victimisation, see [47,48,49]). Swan and colleagues’ [46] research illustrates the normalisation and sometimes glorification of chemsex and chemical submission among men. Yet it is important to note that two of the most prominent male sexual victimisation cases in British judicial history involved GHB.

Firstly is the case of Stephen Port, who drugged, raped and murdered four men, and the other case is that of Reinhard Sinaga, who spiked and raped more than 200 men [50]. The main difference between the cases was thought to be the victim’s sexuality. Ports victims were thought to have consensually consumed GHB, though they were unaware that the offender had purposely increased the dose of the drug to help facilitate his crimes against them. It is important to note that media reports surrounding this case were often lurid in nature, reinforcing prejudice against MSM and Chemsex participation [51]. A recent inquiry found that up to four of his victims’ deaths may have been prevented if not for the homophobic attitudes held by the police officers in charge of investigating their deaths [52]. Sinaga, on the other hand, targeted men regardless of sexuality and used GHB to spike, sexually assault and rape them. Her analysis suggests the media used empathetic and sympathetic language towards the predominately heterosexual victims [50]. This high-profile case put GHB in the public and legal spotlight, with the UK government reclassifying GHB from a class C to class B drug similar to that of ketamine and cannabis, in an effort to quell public and scholarly demands surrounding the drug [51].

Hegemonic masculinities prevail in global communities [53], and sexual crimes committed against men are often seen as less serving as the men embody subordinate masculinities [54]. These victims are seen as challenging hegemonic masculinity; therefore, they are often seen by other men as ‘abnormal’ and ‘deviant’, resulting in their marginalisation [55,56]. This, alongside the negative stigma associated with chemsex [56] and the feminisation of chemical submission victims [57], reduces the likelihood of male victims of drug-facilitated SA coming forward [58]. Studies have repeatedly found that sexuality plays a role in male victim-blaming (VB), with gay men more likely to face secondary victimisation [59,60]. Davies et al. [61] found that heterosexual men often attribute more victim blame towards MSM victims of sexual assault than MSM do. Classical theoretical accounts such as Shaver’s Defensive Attribution Hypothesis suggest that heterosexual men perceive more similarities with other heterosexual men than MSM and are more likely to attribute blame to MSM homosexual men than heterosexual men [62,63]. The literature points to empathy’s role in this hypothesis and how empathy parallels the effects of perceived similarities in victim blame [64,65]. Empathy is recognising and understanding others’ emotions [66]. Research has consistently linked empathy and victim blame attributions [64,67], illustrating that decreased victim empathy coexists with increased blaming attitudes of sexual assault victims [68]. However, an earlier study by Bell et al. [69] failed to associate empathy and victim blame attribution, leading to a lack of consensus in the literature surrounding the exact nature of the relationship.

Although no study, to the authors’ knowledge, has examined the role that victim sexuality plays on empathetic responsiveness towards male victims of sexual violence, Burke et al. [70] found that MSM receives less empathy than heterosexual men. Moreover, Sprankle et al. [67] results indicate that sexual assault victims from minority groups are often more stigmatised and receive less empathy than Caucasian populations (for a review of the experiences of minority male sexual assault victims, see [55]). In a study by Davies et al. [61], the authors found that straight men sympathised significantly more with heterosexual male sexual assault victims than their homosexual counterparts. The authors also found that homosexual men were significantly more likely to be blamed for their victimisation than were heterosexual victims by male participants. Sheridan [71] supports this assertion, finding that men viewed homosexual rape victims as being more ‘compliant’ to their attackers—a prominent and well-established rape myth (for a review of common myths, refer to Willmott et al. [72]). Nevertheless, it can be argued that these studies may well be outdated, with findings that are unlikely to hold true today. Indeed, in recent years, major events may have helped shift public attitudes surrounding MSM and male sexual victimisation, such as the legalisation of gay marriage [73], the growth of ‘PRIDE’ [74] and the ‘MeToo’ movement [75]. 

Contrary to Davies et al. [61] and Sheridan [71], a more recent study by Spiker [76] found that victim blame scores did not differ depending on the victim’s sexuality. As such, this recent study criticises the Minority Stress Theory explanation of MSM drug use and serves as evidence that contradicts previous literature surrounding the association between sexuality, male sexual victimisation and attributions of blame. In fact, the authors conclude that their findings reflect a potential shift in public attitudes towards MSM over the past 20 years. However, it is important to recognise that the authors utilised a sample of just 60 university students. As students often have more liberal attitudes to sexual violence words than the general population [77,78], it is possible that the conclusion reached by Spiker [76] may not extrapolate among more representative community samples.

### Current Study

The current study aims to investigate blame attributions and empathetic responsiveness towards male victims of sexual assault among a community sample of UK men. Specifically, we aim to investigate the role of victim sexuality in the attribution of blame and empathic responsiveness ascribed to male victims of drug-facilitated sexual assault, thereby reexamining the findings of Spiker [76] and Davies et al. [61] and dominant theorical explanations therein. To this end, we hypothesise that there will be a significant difference in victim blame attribution scores and empathy ratings as a direct consequence of the victim’s reported sexuality and engagement in drug consumption. We hypothesise that victims in the chemsex conditions will attract the highest victim blame attribution scores and the lowest victim empathy ratings, according to the existing male rape myth literature. Specifically, we hypothesise an interaction between sexuality and victim blame and empathy ratings, with homosexual men in the MSM and chemsex conditions attracting significantly higher victim blame attributions and significantly lower victim empathy scores than the heterosexual victims in these same scenarios.

## 2. Methods

### 2.1. Study Design

A quantitative independent between-groups experimental design was adopted, utilising six vignette variants of the same male-sexual assault scenario (Appendix A). Two independent variables were manipulated: (1) victim sexuality (*heterosexual* vs. *homosexual*) and (2) drug consumption during the sexual assault (*chemsex*, *chemical submission*, *no drugs*) to examine the impact on two dependent variables: (1) blame attribution scores and (2) victim empathy scores.

### 2.2. Participants

Participants were recruited using a combination of opportunistic and snowball sampling through an electronic poster distributed across various social media platforms (i.e., Facebook, LinkedIn and Twitter/X) that included a web link to the Qualtrics data collection platform. Social media were used in an effort to reach a wider and more diverse proportion of the general public than simply recruiting students on campus. Priori tests were performed using G*Power software to determine the study’s sample required size based on the conditions and variables under scrutiny. To achieve the desired power of 0.8 using an alpha of 0.05 and a medium effect size of 0.25, a sample of 119 participants was required. In total, 154 participants were recruited, though after removing participants (as discussed below), the final sample retained for analysis was 141. Due to the topic’s sensitive nature, exclusion criteria meant that those under the age of 18 years, non-UK citizens and participants who did not identify as male were not able to participate. This led to five participants being removed from the final dataset prior to analysis who did not meet these criteria. As the study relied on participants thoroughly reading and digesting details explained in a written vignette, validation and engagement check questions were asked before capturing scores on the dependent variables. A further three participants incorrectly answered these questions, and their data were therefore removed prior to analysis. Finally, based on an examination of the spread of participant scores, a further five participants were identified as extreme outliers and removed from the final dataset. Among the final sample who provided their age, participants ranged from 18–77 years old (*M* = 39.84, *SD* = 14.12). Three participants did not disclose their age, though their data were retained, given that they confirmed that they were over the age of 18. Regarding occupation, 63% of the sample reported being in full-time employment, 6% were employed part-time, 12% were full-time students and the remaining 19% were retired or ‘other’. For sexuality, 62% of the sample were heterosexual men (n = 88), and the remaining 38% were men who have sex with men (MSM) (n = 53) (25% gay; 11% bisexual; 2% queer). 

### 2.3. Procedure and Materials

After participants clicked the study link online, they were first presented with a participant information sheet that outlined the nature and purpose of the study. Next, the participants were shown a consent form that they were required to read and attest to before being able to take part. Those who consented were then asked to complete a demographic questionnaire which collected data regarding gender, sexuality, age and employment to establish that the inclusion/exclusion criteria had been met. Next, participants were randomly assigned to one of the six experimental conditions where they were asked to read a vignette involving a male SA scenario (see the appendices for all vignettes). Vignettes were developed using those included in Spiker’s [76] study, alongside qualitative data regarding chemsex sexual assault in Bourne et al.’s [79] study. Methodological research indicates that participants are more likely to skip or skim descriptive text included in written vignettes [73], especially when making judgements about culpability in sexual assault scenarios compared to more engaging case presentation formats [72]; therefore, to reduce the influence of disengagement on participant responses, two questions unique to the facts of each vignette were asked (e.g., *where did Chris first meet Andy?*). Finally, participants were then asked to complete two questionnaires (described below), where they expressed their attribution blame and empathy towards those involved in the scenario they had just read. After completing the study, participants were provided with a study debrief outlining additional information about the aims of the study, the process of withdrawing their data should they want to (note: no participants did), contact details for the researchers and free independent support services. 

### 2.4. Measures

***Male Rape Victim and Perpetrator Blaming (MRVPB)*** ***Scale*** [80]. The MRVPB was used to measure the participants’ victim blame attributions towards the victim described in the vignettes. The scale was slightly modified simply to match the names of the victim and perpetrator in the current study’s vignettes. While Spiker [76] utilised the Male Rape Myths scale [MRMS] [81] to measure such attitudes, the MRVPB, given the items included are more contemporary and were generated following more rigorous psychometric testing than the MRMS. A five-point Likert rating scale was adopted where 1 = ‘Not at all’ and 5 = ‘Extremely/Totally’. To prevent forced-choice questionnaire bias, a ‘Moderately’ mid-point option was included. Six of the scale items were reverse-scored, meaning a maximum individual score of 41. Higher scores equate to greater victim blame attribution. Scale reliability (a = 0.72). An item example of the questionnaire is: “*In your opinion, was Andy sexually provocative?*” 

***Victim Blame and Empathy Scale (VBE)*** [82]. Three items of the Victim Blame and Empathy scale (VBE) were utilised to assess empathy towards male victims of sexual assault. Again, a five-point Likert rating scale was adopted where 1 = ‘Not at all’ and 5 = ‘Extremely/Totally’. The scale has a low-reliability score (a = 0.41); however, this is not uncommon amongst scales that contain a small number of items [83]. Despite the reliability score, the scale was utilised as previous literature has used these scale items to measure empathetic responsiveness towards victims of sexual violence [67]. Possible scores range from 5 to 15. With higher scores indicative of increased empathy expressed towards the victim in the vignette. An example of an item on the questionnaire is: “*How badly did you feel for the victim?*”

***Participant Sexuality.*** Participants’ responses to the sexuality question were categorised for the purpose of analysis. Those who identified as ‘gay’, ‘bisexual’, or ‘queer’ were grouped into the ‘MSM’ group and those who identified as ‘straight’ were grouped into the ‘heterosexual’ group. 

### 2.5. Ethical Considerations

Ethical approval for the study was obtained from **BLANKED FOR PEER-REVIEW** (approval ID 43452; approved June 2022). The study was conducted following the British Psychological Society (BPS) guidelines for human research [84] and in accordance with institutional ethics policies. Moreover, all participants provided informed consent, free from deception, were informed upfront about the sensitive nature of the study and their rights to withdraw, alongside the use of a screening question to ensure that participants were aware of the study’s focus on male sexual behaviour and assault before participation began. Participants were reminded that their responses were anonymous and provided with a unique identification code, which allowed them to withdraw their data at any point of the study or after completion until the date stated. Information on free and impartial support services was provided at the onset and end of the study.

### 2.6. Analytical Procedures

Data screening took place prior to the MANOVA analysis. No input errors or missing data were found. Skewness and Kurtosis calculations showed that the data is not skewed as groups fell within the accepted value (−1.96 and 1.96) [85]. Z-scores suggest that five outliers were problematic as they failed to satisfy the acceptable +/−3 [86]. Additionally, comparing the Mahalanobis distance values to the chi-square distribution, none of these outliers was identified as problematic. As per Aguinis et al. [87], the five outliers were removed. After outliers were removed, the data followed the normal Q–Q plots and the data balanced above and below the detrended Q–Q plots. The assumption that there is an absence of multicollinearity was tested, and as *r* = −0.38, the assumption was met [88]. Levene’s test showed that the variances of the groups were equal on both the MRVPB scale, *F*(2, 142) = 1.03, *p* = 0.43 and the VBE scale, *F*(2, 142) = 1.06, *p* = 0.40. As *p* > 0.05, it is assumed that there is equality of variance within the samples (homogeneity of variance) [89]. Box’s test was used to assess the homogeneity of variance-covariance matrices, and the result was non-significant (44.21 *p* < 0.0001) [90]. As assumptions were met, interpretation used Wilk’s lambda. 

## 3. Results

### 3.1. Descriptive Statistics

Descriptive statistics, illustrating the means (M) and standard deviation (SD) for the Male Rape Victim and Perpetrator Blaming (MRVPB) scale scores and each condition, are presented in Table 1, while Table 2 presents the descriptive statistics for the Victim Blame and Empathy (VBE) scale scores. Interpretation of the means supports the hypothesis that straight men attributed the highest levels of victim blame (M = 22.19, SD = 5.68) and lowest levels of victim empathy (M = 7.60, SD = 2.22) overall. Contrary to our hypothesis, straight men faced the highest levels of victim blame (M = 23.00, SD = 5.61) and lowest levels of victim empathy (M = 7.60, SD = 2.52) in the chemsex condition, and attracted the highest empathy levels when in the chemical submission condition (M = 8.38, SD = 2.11). Likewise, in that chemsex condition, the highest victim blame scores were observed (M = 22.54, SD = 5.77), and the lowest victim empathy scores (M = 7.19, SD = 2.17). An examination of descriptive statistics alone indicates that straight male participants attributed the most victim blame (M = 23.23, SD = 5.10) and the least empathy (M = 7.15, SD = 1.77) to heterosexual victims in the chemsex condition.

### 3.2. Inferential Statistics

Using Wilks’ lambda, there was a significant effect of participant sexuality on victim blame and empathetic responsiveness towards victims, *V* = 0.07, *F*(2, 128) = 4.53, *p* = 0.013, η^2^ = 0.066. However, separate univariate tests on the outcome variables revealed non-significant attributions for victim blame scores, *F*(1, 129) = 3.45, *p* = 0.066, η^2^ = 0.026, though significant findings for empathetic responsiveness *F*(1, 129) = 8.41, *p* = 0.004, η^2^ = 0.061. As such, a non-significant effect was observed between conditions (victim sexuality and drug-facilitated sexuality assault), *V* = 0.95, *F*(10, 258) = 0.64, *p* = 0.780, η^2^ = 0.024. Additionally, there was a non-significant interaction between conditions and participant sexuality, *V* = 0.94, *F*(10, 256) = 0.75, *p* = 0.674 η^2^ = 0.028.

## 4. Discussion

The current study aimed to examine victim blame and empathy assessments towards male drug-facilitated sexual assault among a UK community sample of men. While no interaction was found between sexuality and type of drug-facilitated sexual assault, significant differences were observed in victim empathy scores between participants of different sexualities. The first hypothesis is partly supported in that straight men were found to be significantly less likely to empathise with sexual assault victims compared to MSM participants. However, no such differences were observed in victim blame attributions more broadly. This finding appears to somewhat support the conclusions drawn by Spiker [76], with negative attitudes towards male victims of assault seeming to be in decline among the current sample. With no significance found, our findings contradict those of Davies et al. [61]. An explanation for this finding may be that stigma toward sexual assault victims has begun to decline or greater understanding of the experiences of male victims is emerging [75], resulting in a reduction in victim blame attributions. Nevertheless, as MSM and sexual assault victims often embody subordinate masculinity [54], straight men may view themselves as more ‘hegemonic’. As a result, they possibly cannot see themselves in a scenario where they too could experience sexual violence, especially as the vignettes used a male perpetrator, therefore participants are perhaps less likely to empathise with the victim than MSM, who may recognise the circumstance within which the victim finds themself.

No significant main effect was found between experimental conditions and blame/empathy ratings. While it was hypothesised that MSM would likely attract the greatest victim blame and least empathetic responsiveness, this finding was not supported in the current data. The finding accords with Spiker [76] though it contradicts most prior literature regarding the role that sexuality may have on victim blame attributions [60] and victim empathy [70]. Despite no significant differences being found, an interpretation of mean scores appears to contradict this hypothesis, with heterosexual victims in the chemsex condition attracting the highest levels of victim blame and the lowest empathy ratings. However, caution is needed here, given the lack of significant differences emerging overall. Indeed, empathy ratings suggest that straight victims in the chemical submission scenario attracted the greatest empathy overall. Straight men participating in chemsex and voluntarily taking GHB breaks the narrative of ‘just MSM participate in chemsex and take drugs’, hence the possible reasons for higher victim blame and decreased empathy overall. 

On the other hand, straight men being victims of chemical submission does not conform to popular narratives; therefore, given the perceived rarity, it may have been seen as more shocking and abnormal, similar to the media coverage and focus on heterosexual victims of the Sinaga case [50]. Next, although no significance was observed between conditions overall, the mean scores suggest that those who read a variant of the chemsex scenario attributed more victim blame and less empathy than the chemical submission scenario. This was perhaps expected due in part to the negative stigma associated with chemsex, (as seen in media coverage of the Port case [91]) when compared to the Sinaga case that involved chemical submission. Overall, the findings indicate that regardless of sexuality or drug-facilitated sexual assault variation, mean scores for victim blame and empathy sit around the mid-range of the scales. This suggests that most participants selected the ‘Neither Agree nor Disagree’ option. The most probable explanation is the lack of knowledge surrounding chemsex, GHB and chemical submission in the context of male sexual assault. As such, participants may have found it more difficult to formulate an opinion compared to if the victim was female or no drugs were involved. Indeed, prior studies examining public attitudes towards rape victim compensation entitlement suggest some uncertainty among respondents between unfamiliar assault scenarios and when the circumstances are considered somewhat in the abstract [92]. Future research should seek to examine participants’ pre-existing knowledge or provide more detailed information regarding the circumstances’ scrutiny. 

### 4.1. Limitations and Future Recommendations

Firstly, as self-reported data were used, there is a chance that participants will intentionally or unintentionally misrepresent their responses in order to give a false impression that is not representative of their actual perceptions. Despite this potential limitation, this is, of course, a limitation in a wealth of similar cross-sectional studies gathering data from the public about such topics. Future research may benefit from capturing and examining qualitative data to help provide a holistic view of the interaction between sexuality, the scenarios employed depicting variants of drug-facilitated sexual assault, and participant empathy/blame attributions. Indeed, recent research has utilised such an approach in seeking to better understand contemporary reasons why rape victim-survivors choose not to report [93], the mental health consequences of sexual assault [94] and non-abusive men’s perceptions of how to tackle male partner violence [95].

Further concerns may be raised regarding the empathy items and measurement scale used. Moreover, given the low internal reliability score of the Victim Blame and Empathy Scale (VBE) items included, future research ought to make use of an alternative, more psychometrically robust victim empathy scale (for more substantive analysis of victim empathy tools see Debowska and colleagues [65]). Next, while the sample made use of a community sample, this sample was both small and non-proportionally representative of the UK population. Age is particularly important in this domain considering that chemsex is a relatively recent phenomenon [10] and is on the rise amongst younger populations, regardless of sexuality [14]. Future research should seek to adopt a more systematic random sample of the UK public before robust conclusions about participant attitudes towards these crime types can be understood. 

### 4.2. Study Implications

This study’s findings support results obtained by Spiker [76] in that they indicate a potential lack of understanding surrounding chemsex and drug-facilitated sexual assault among MSM. Policymakers rely on public support to fund, change and enact new legislation, as seen in the case of Singa and the reclassification of GHB. As such, public uncertainty illustrates here the need for future research and psychoeducation to highlight the consequences of GHB use, chemsex participation and male sexual victimisation in chemical submission instances. In turn, this will help reduce the stigma, allowing victims to come forward and seek justice, treatment or wider support where needed. Indeed, a wide range of studies that find continued evidence of public uncertainty or indeed problematic endorsement of myths and falsehoods surrounding a wide range of sexual offences also endorse the use of widespread mainstream educational interventions [96,97,98,99,100]. 

At the time of writing, limited research has investigated public attitudes towards chemsex. Despite the aforementioned limitations of this work, the study therefore offers some foundational insight into public perceptions towards this form of drug-facilitated sexual assault. As those in the chemsex scenario did encounter the greater victim blame and lowest levels of empathy, it is clear there are negative attitudes associated with chemsex that require tackling if victim-survivors of violence in these circumstances are to feel confident in disclosing their experiences to loved ones and authorities. This is especially important considering the civic function that the public serves as jurors [101,102], where evidence suggests pre-trial knowledge and attitudes can have an important prejudicial influence on trial outcomes [103,104]. Future research should, therefore, look to make use of the current study vignettes and real case scenarios in examining the extent to which jury bias and decision-making can be considered fair and impartial in MSM cases where chemsex is present. 

## 5. Conclusions

To conclude, this study found a significant difference between participant sexuality and victim empathetic responsiveness towards male victims of sexual assault. However, elsewhere, the study failed to discern any difference in victim-blaming attitudes between the experimental conditions. The findings suggest that those in the chemsex condition faced the highest levels of victim blame and lowest empathy as expected. However, no interaction was found based upon victim or respondent sexuality with types of drug-facilitated sexual assault. Nevertheless, to the authors’ knowledge, this is the first study to investigate attitudes towards chemsex and GHB in the context of the victim blame and empathy literature. The findings may have raised more questions than answers, though the study serves as the foundation from which further research can explore public and thus, juror perceptions towards chemsex in male sexual assault. 

## Figures and Tables

**Table 1:** Descriptive statistics showing the means and standard deviations of victim-blaming attitudes towards male sexual assault victims depending on participant sexuality, the drug-facilitated sexual assault and victim sexuality.

Measure		Participant’s Sexuality					
		Straight		MSM		Average Score	
		M	SD	M	SD	M	SD
Chemsex							
	Straight	23.23	5.10	22.67	6.58	23.00	5.61
	MSM	22.95	6.34	19.71	4.20	22.08	5.94
					Average	22.54	5.77
Chemical Submission							
	Straight	22.84	4.43	20.70	5.00	22.10	4.65
	MSM	22.33	7.41	19.82	5.00	21.13	6.36
					Average	21.62	5.01
Controlled Group							
	Straight	21.50	7.13	18.88	5.00	20.45	6.35
	MSM	19.62	3.31	19.88	4.29	19.71	6.35
					Average	20.08	4.98
Average Score		22.19	5.68	20.32	5.00	21.49	5.49

Note: M—mean; SD—standard deviation; MSM—men who have sex with men.

**Table 2:** Descriptive Statistics showing the means and standard deviations of empathetic attitudes towards male sexual assault victims depending on participant sexuality, the drug-facilitated sexual assault and victim sexuality.

Measure		Participant’s Sexuality					
		Straight		MSM		Average Score	
		M	SD	M	SD	M	SD
Chemsex							
	Straight	7.15	1.77	8.22	3.35	7.60	2.52
	MSM	7.21	1.44	9.57	1.72	7.85	1.83
					Average	7.19	2.17
Chemical Submission							
	Straight	8.32	2.40	8.50	1.51	8.38	2.11
	MSM	7.25	1.91	8.73	1.85	8.00	2.00
					Average	8.12	2.05
Controlled Group							
	Straight	8.25	2.73	8.13	2.17	8.20	2.46
	MSM	7.31	1.75	8.88	2.70	7.90	2.33
					Average	8.05	2.35
Average Score		7.60	2.04	8.64	2.22	7.99	2.16

Note: M—mean; SD—standard deviation; MSM—men who have sex with men.

## Data Availability

The data is available via email request to the authors.

## Appendix A

### Appendix A.1. Vignettes

#### Appendix A.1.1. Vignette 1—Chemsex/Straight Male

Andy is an 18-year-old male who has recently moved from Kent to Manchester for university. As he is new to the area and single, he has started to use popular dating sites such as Tinder and Bumble to meet women for casual sex, dates and make new friends. In his dating profile, he makes it clear that he is not looking for anything serious. Late Monday evening Andy was approached on Bumble by a blank profile with the headline ‘Fun?’. After chatting for a while, it was clear to Andy that the user was also looking for casual sex and nothing serious. Andy noticed that the user had ‘PnP’ in their bio and asked what it meant. The user explained that it stood for ‘Party and Play’ and that he often takes the drug ‘g’ (GHB) to enhance “the feeling and experience”. Andy explained how he had never taken this drug before and, although cautious at first, was open to taking it. The user sent Andy a picture of himself, and to Andy’s surprise, it was a guy who looked around the age of 26/27 years of age who introduced himself as ‘Chris’. Andy explained how he had never done anything sexual with a guy before but was open to new experiences. The two exchanged numbers and agreed to meet on Thursday. Thursday evening came, and Andy received a text message from Chris with his home address asking, “What time are you coming then?” Although Andy was nervous, he was feeling rather lonely in this new city and thought this was a perfect opportunity to meet new people. They agreed on 10 pm. Andy arrived at 9:45 pm and stood on the street corner, building the courage to go to the door. When he finally arrived, Chris opened the door and led Andy to the living room. After chatting for a while, Chris sensed Andy was nervous and suggested they “put a film on for a bit”. Halfway through the film, Chris offered Andy a drink, to which Andy agreed. Chris walked back into the living room, saying, “It’s Diet Coke, if that’s okay?” Andy agreed. Chris explained that the drink had ‘g’ in and that he was about to have “the best sex in his life”. The pair continued to chat and watch the film. After nearly finishing his drink, Andy started to feel dizzy and nauseous. He recalls feeling as if he was drunk. Andy then passed out. He woke up confused and dazed; he noticed that Chris was performing oral sex on him. Andy panicked and tried to push Chris off. Chris laughed and tried to reassure Andy, saying that “It always happens; I probably gave you too much ‘g’”. Andy was in shock and froze while Chris continued to perform oral sex. Chris then headed upstairs and told Andy to join him. As Chris left the room, Andy grabbed his belongings, got dressed and left. The next day, Andy does not remember the events of the night and that even the walk home is a fuzzy memory. He had received a text message from Chris asking where he had gone and how he had such a great time. Andy is still in shock and is angry at Chris. He expresses his anger in a text message asking how he could do something like that. Chris replied, confused at what he had done wrong. Andy blocked Chris and is unsure of what to do next.

#### Appendix A.1.2. Vignette 2—Chemsex/Gay Male

Andy is an 18-year-old male who has recently moved from Kent to Manchester for university. As he is new to the area and single, he has started to use popular dating sites such as Tinder and Grindr to meet men for casual sex, dates and make new friends. In his dating profile, he makes it clear that he is not looking for anything serious. Late Monday evening, Andy was approached on Grindr by a blank profile with the headline ‘Fun?’. After chatting for a while, it was clear to Andy that the user was also looking for casual sex and nothing serious. Andy noticed that the user had ‘PnP’ in their bio and asked what it meant. The user explained that it stood for ‘Party and Play’ and that he often takes the drug ‘g’ (GHB) to enhance “the feeling and experience”. Andy explained how he had never taken this drug before and, although cautious at first, was open to taking it. The user sent Andy a picture of himself, and it was a guy who looked around the age of 26/27 years of age who introduced himself as ‘Chris’. The two exchanged numbers and agreed to meet on Thursday. Thursday evening came, and Andy received a text message from Chris with his home address asking, “What time are you coming then?”. Although Andy was nervous, he was feeling rather lonely in this new city and thought this was a perfect opportunity to meet new people. They agreed on 10 pm. Andy arrived at 9:45 pm and stood on the street corner, building the courage to go to the door. When he finally arrived, Chris opened the door and led Andy to the living room. After chatting for a while, Chris sensed Andy was nervous and suggested they “put a film on for a bit”. Halfway through the film, Chris offered Andy a drink, to which Andy agreed. Chris walked back into the living room, saying, “it’s Diet Coke, if that’s okay?” Andy agreed. Chris explained that the drink had ‘g’ in and that he was about to have “the best sex in his life”. The pair continued to chat and watch the film. After nearly finishing his drink, Andy started to feel dizzy and nauseous. He recalls feeling as if he was drunk. Andy then passed out. He woke up confused and dazed; he noticed that Chris was performing oral sex on him. Andy panicked and tried to push Chris off. Chris laughed and tried to reassure Andy, saying that “It always happens; I probably gave you too much ‘g’”. Andy was in shock and froze while Chris continued to perform oral sex. Chris then headed upstairs and told Andy to join him. As Chris left the room, Andy grabbed his belongings, got dressed and left. The next day, Andy does not remember the events of the night and that even the walk home is a fuzzy memory. He had received a text message from Chris asking where he had gone and how he had such a great time. Andy is still in shock and is angry at Chris. He expresses his anger in a text message, asking how he could do something like that. Chris replied, confused at what he had done wrong. Andy blocked Chris and is unsure of what to do next.

#### Appendix A.1.3. Vignette 3—Spiked/Straight Male

Andy is an 18-year-old male who has recently moved from Kent to Manchester for university. As he is new to the area and single, he has started to use popular dating sites such as Tinder and Grindr to meet men for casual sex, dates and make new friends. In his dating profile, he makes it clear that he is not looking for anything serious. Late Monday evening, Andy was approached on Grindr by a blank profile with the headline ‘Fun?’. After chatting for a while, it was clear to Andy that the user was also looking for casual sex and nothing serious. Andy noticed that the user had ‘PnP’ in their bio and asked what it meant. The user explained that it stood for ‘Party and Play’ and that he often takes the drug ‘g’ (GHB) to enhance “the feeling and experience”. Andy explained how he had never taken this drug before and, although cautious at first, was open to taking it. The user sent Andy a picture of himself, and it was a guy who looked around the age of 26/27 years of age who introduced himself as ‘Chris’. The two exchanged numbers and agreed to meet on Thursday. Thursday evening came, and Andy received a text message from Chris with his home address asking, “What time are you coming then?”. Although Andy was nervous, he was feeling rather lonely in this new city and thought this was a perfect opportunity to meet new people. They agreed on 10 pm. Andy arrived at 9:45 pm and stood on the street corner, building the courage to go to the door. When he finally arrived, Chris opened the door and led Andy to the living room. After chatting for a while, Chris sensed Andy was nervous and suggested they “put a film on for a bit”. Halfway through the film, Chris offered Andy a drink, to which Andy agreed. Chris walked back into the living room, saying, “It’s Diet C that’s okay?” Andy agreed. Chris explained that the drink had ‘g’ in and that he was about to have “the best sex in his life”. The pair continued to chat and watch the film. After nearly finishing his drink, Andy started to feel dizzy and nauseous. He recalls feeling as if he was drunk. Andy then passed out. He woke up confused and dazed; he noticed that Chris was performing oral sex on him. Andy panicked and tried to push Chris off. Chris laughed and tried to reassure Andy, saying that “It always happens; I probably gave you too much ‘g’”. Andy was in shock and froze while Chris continued to perform oral sex. Chris then headed upstairs and told Andy to join him. As Chris left the room, Andy grabbed his belongings, got dressed and left. The next day, Andy does not remember the events of the night and that even the walk home is a fuzzy memory. He had received a text message from Chris asking where he had gone and how he had such a great time. Andy is still in shock and is angry at Chris. He expresses his anger in a text message, asking how he could do something like that. Chris replied, confused at what he had done wrong. Andy blocked Chris and is unsure of what to do next.

#### Appendix A.1.4. Vignette 4—Spiked/Gay Male

Andy is an 18-year-old male who has recently moved from Kent to Manchester for university. As he is new to the area and single, he has started to use popular dating sites such as Tinder and Grindr to meet men for casual sex, dates and make new friends. In his dating profile, he makes it clear that he is not looking for anything serious. Late Monday evening, Andy was approached on Grindr by a blank profile with the headline ‘Fun?’. After chatting for a while, it was clear to Andy that the user was also looking for casual sex and nothing serious. Andy noticed that the user had ‘PnP’ in their bio and asked what it meant. The user explained that it stood for ‘Party and Play’ and that he often takes the drug ‘g’ (GHB) to enhance “the feeling and experience”. Andy explained how he had never taken this drug before and, although cautious at first, was open to taking it. The user sent Andy a picture of himself, and it was a guy who looked around the age of 26/27 years of age who introduced himself as ‘Chris’. The two exchanged numbers and agreed to meet on Thursday. Thursday evening came, and Andy received a text message from Chris with his home address asking, “What time are you coming then?”. Although Andy was nervous, he was feeling rather lonely in this new city and thought this was a perfect opportunity to meet new people. They agreed on 10 pm. Andy arrived at 9:45 pm and stood on the street corner, building the courage to go to the door. When he finally arrived, Chris opened the door and led Andy to the living room. After chatting for a while, Chris sensed Andy was nervous and suggested they “put a film on for a bit”. Halfway through the film, Chris offered Andy a drink, to which Andy agreed. Chris walked back into the living room, saying, “it’s Diet Coke, if that’s okay?” Andy agreed. Chris explained that the drink had ‘g’ in and that he was about to have “the best sex in his life”. The pair continued to chat and watch the film. After nearly finishing his drink, Andy started to feel dizzy and nauseous. He recalls feeling as if he was drunk. Andy then passed out. He woke up confused and dazed; he noticed that Chris was performing oral sex on him. Andy panicked and tried to push Chris off. Chris laughed and tried to reassure Andy, saying that “It always happens; I probably gave you too much ‘g’”. Andy was in shock and froze while Chris continued to perform oral sex. Chris then headed upstairs and told Andy to join him. As Chris left the room, Andy grabbed his belongings, got dressed and left. The next day, Andy does not remember the events of the night and that even the walk home is a fuzzy memory. He had received a text message from Chris asking where he had gone and how he had such a great time. Andy is still in shock and is angry at Chris. He expresses his anger in a text message, asking how he could do something like that. Chris replied, confused at what he had done wrong. Andy blocked Chris and is unsure of what to do next.

#### Appendix A.1.5. Vignette 5—Controlled Group—No Drugs/Straight Male

Andy is an 18-year-old male who has recently moved from Kent to Manchester for university. As he is new to the area and single, he has started to use popular dating sites such as Tinder and Grindr to meet men for casual sex, dates and make new friends. In his dating profile, he makes it clear that he is not looking for anything serious. Late Monday evening, Andy was approached on Grindr by a blank profile with the headline ‘Fun?’. After chatting for a while, it was clear to Andy that the user was also looking for casual sex and nothing serious. Andy noticed that the user had ‘PnP’ in their bio and asked what it meant. The user explained that it stood for ‘Party and Play’ and that he often takes the drug ‘g’ (GHB) to enhance “the feeling and experience”. Andy explained how he had never taken this drug before and, although cautious at first, was open to taking it. The user sent Andy a picture of himself, and it was a guy who looked around the age of 26/27 years of age who introduced himself as ‘Chris’. The two exchanged numbers and agreed to meet on Thursday. Thursday evening came, and Andy received a text message from Chris with his home address asking, “What time are you coming then?”. Although Andy was nervous, he was feeling rather lonely in this new city and thought this was a perfect opportunity to meet new people. They agreed on 10 pm. Andy arrived at 9:45 pm and stood on the street corner, building the courage to go to the door. When he finally arrived, Chris opened the door and led Andy to the living room. After chatting for a while, Chris sensed Andy was nervous and suggested they “put a film on for a bit”. Halfway through the film, Chris offered Andy a drink, to which Andy agreed. Chris walked back into the living room, saying, “it’s Diet Coke, if that’s okay?” Andy agreed. Chris explained that the drink had ‘g’ in and that he was about to have “the best sex in his life”. The pair continued to chat and watch the film. After nearly finishing his drink, Andy started to feel dizzy and nauseous. He recalls feeling as if he was drunk. Andy then passed out. He woke up confused and dazed; he noticed that Chris was performing oral sex on him. Andy panicked and tried to push Chris off. Chris laughed and tried to reassure Andy, saying that “It always happens; I probably gave you too much ‘g’”. Andy was in shock and froze while Chris continued to perform oral sex. Chris then headed upstairs and told Andy to join him. As Chris left the room, Andy grabbed his belongings, got dressed and left. The next day, Andy does not remember the events of the night and that even the walk home is a fuzzy memory. He had received a text message from Chris asking where he had gone and how he had such a great time. Andy is still in shock and is angry at Chris. He expresses his anger in a text message, asking how he could do something like that. Chris replied, confused at what he had done wrong. Andy blocked Chris and is unsure of what to do next.

#### Appendix A.1.6. Vignette 6—Control Group—No Drugs/Gay Male

Andy is an 18-year-old male who has recently moved from Kent to Manchester for university. As he is new to the area and single, he has started to use popular dating sites such as Tinder and Grindr to meet men for casual sex, dates and make new friends. In his dating profile, he makes it clear that he is not looking for anything serious. Late Monday evening, Andy was approached on Grindr by a blank profile with the headline ‘Fun?’. After chatting for a while, it was clear to Andy that the user was also looking for casual sex and nothing serious. Andy noticed that the user had ‘PnP’ in their bio and asked what it meant. The user explained that it stood for ‘Party and Play’ and that he often takes the drug ‘g’ (GHB) to enhance “the feeling and experience”. Andy explained how he had never taken this drug before and, although cautious at first, was open to taking it. The user sent Andy a picture of himself, and it was a guy who looked around the age of 26/27 years of age who introduced himself as ‘Chris’. The two exchanged numbers and agreed to meet on Thursday. Thursday evening came, and Andy received a text message from Chris with his home address asking, “What time are you coming then?”. Although Andy was nervous, he was feeling rather lonely in this new city and thought this was a perfect opportunity to meet new people. They agreed on 10 pm. Andy arrived at 9:45 pm and stood on the street corner, building the courage to go to the door. When he finally arrived, Chris opened the door and led Andy to the living room. After chatting for a while, Chris sensed Andy was nervous and suggested they “put a film on for a bit”. Halfway through the film, Chris offered Andy a drink, to which Andy agreed. Chris walked back into the living room, saying, “it’s Diet Coke, if that’s okay?” Andy agreed. Chris explained that the drink had ‘g’ in and that he was about to have “the best sex in his life”. The pair continued to chat and watch the film. After nearly finishing his drink, Andy started to feel dizzy and nauseous. He recalls feeling as if he was drunk. Andy then passed out. He woke up confused and dazed; he noticed that Chris was performing oral sex on him. Andy panicked and tried to push Chris off. Chris laughed and tried to reassure Andy, saying that “It always happens; I probably gave you too much ‘g’”. Andy was in shock and froze while Chris continued to perform oral sex. Chris then headed upstairs and told Andy to join him. As Chris left the room, Andy grabbed his belongings, got dressed and left. The next day, Andy does not remember the events of the night and that even the walk home is a fuzzy memory. He had received a text message from Chris asking where he had gone and how he had such a great time. Andy is still in shock and is angry at Chris. He expresses his anger in a text message, asking how he could do something like that. Chris replied, confused at what he had done wrong. Andy blocked Chris and is unsure of what to do next.
